# Preparation and Optimization of Ibrutinib-Loaded Nanoliposomes Using Response Surface Methodology

**DOI:** 10.3390/polym14183886

**Published:** 2022-09-17

**Authors:** Fareeaa Ashar, Umme Hani, Riyaz Ali M. Osmani, Syed Mohammed Kazim, S. Selvamuthukumar

**Affiliations:** 1 Department of Pharmacy, Annamalai University, Annamalai Nagar 608002, India; 2 Department of Pharmaceutics, College of Pharmacy, King Khalid University, Abha 62529, Saudi Arabia; 3 Department of Pharmaceutics, JSS College of Pharmacy, JSS Academy of Higher Education and Research, SS Nagara, Mysuru 570015, India; 4 Nizam Institute of Pharmacy, Deshmukhi 508284, India

**Keywords:** nanoliposomes, ibrutinib, encapsulation efficiency, stability, optimization

## Abstract

The main aim of this study was to optimize the formulation and process variables for the preparation of ibrutinib nanoliposomes and to evaluate the stability of nanoliposomes. The influence of four formulations and process parameters, namely, the phosphatidylcholine-to-cholesterol ratio (A), conc. of ibrutinib (B), sonication time (C), and stirring time (D) on the drug encapsulation efficiency (Y_1_) and particle size (Y_2_) of ibrutinib nanoliposomes were investigated by using response surface methodology. Reverse-phase evaporation was used to prepare ibrutinib nanoliposomes. Twenty-nine trial experiments were performed as per the design and the response parameters were noted. Multiple linear regression analysis was used to assess each response parameter. The effect of each factor on the response parameters was depicted using perturbation, response surface, and contour plots. A numerical optimization technique was used to estimate the optimum process parameters to obtain the desired responses. Ibrutinib nanoliposomes prepared under optimal conditions were evaluated for stability at a different temperature, pH, and sonication time. It is evident from the results that the phosphatidylcholine-to-cholesterol ratio (A) was the major factor influencing the encapsulation efficiency. All the factors were found to have noteworthy influences on particle size. A statistical evaluation provided the information about the individual and interactive effects of independent factors on the response parameters in order to obtain optimum experimental conditions that lead to preparing nanoliposomes with improved characteristics. The optimum level of the independent variables was phosphatidylcholine:cholesterol (6.76:1), ibrutinib concentration (2 mg/mL), sonication time (15.13 min), and stirring time (45 min). At optimal conditions, Y_1_ and Y_2_ were found to be 90.76 ± 1.56% and 208.24 ± 3.16 nm, respectively. The ibrutinib nanoliposomes were found to be stable both in simulated gastric and intestinal fluids at 37 °C for 6 h. At elevated conditions of temperature and pH, the prepared nanoliposomes were found to be unstable. Sonication for shorter periods resulted in decreased particle size, whereas longer periods can be helpful for ultrasound-assisted drug delivery. The closeness between the obtained results and predicted results indicates the reliability of the optimization technique for the preparation of ibrutinib nanoliposomes.

## 1. Introduction

Ibrutinib (IB) is a specific inhibitor of the enzyme Bruton’s tyrosine kinase (BTK), used for treatment of B-cell lymphoma. Ibrutinib acts by forming covalent bonds with cysteine residue C481 at phosphorylation sites of BTK, which leads to permanent inactivation and block signals that stimulate uncontrolled growth and division of malignant cells [1,2]. Apart from BTK, IB has been extensively described to block the activation of other kinases such as B lymphocyte kinase (BLK), hematopoietic cell kinase (HCK), interleukin-2 (IL-2) inducible T-cell kinase (ITK), Janus protein tyrosine kinases (JAK3), Tyrosine protein kinase (TEC), TEC family kinase (TFK), and especially erythroblastic leukemia viral oncogene homologue (ERBB) receptor family, indicating the potential of IB for further exploitation for the treatment of multiple types of tumors in the future [3,4,5,6,7]. It has also received regulatory approval in some countries for certain conditions such as mantle cell lymphoma, chronic lymphocytic leukaemia, and Waldenstrom’s macroglobulinemia. Ibrutinib is reported to have a very low bioavailability (2.9%) as it is practically insoluble in water with a mole fraction solubility of about 1.43 × 10^−7^ at room temperature. The partition coefficient value of ibrutinib is 3.74. It is a weak base with a pH-dependent solubility. It is slightly soluble at a pH of 1.2 and is insoluble at a pH of 3 to 8. The pH-dependent solubility resulted in low bioavailability and impede its in vivo antitumor effect after oral administration [8]. Furthermore, the increased pH probably causes the drug to precipitate when ibrutinib is transported from the stomach to the intestine. In addition, ibrutinib is reported to undergo extensive hepatic clearance. Due to the first pass effect and poor bioavailability, it is commercially developed in very high doses (140 mg per capsule), which results in severe gastrointestinal adverse effects [9,10,11,12,13,14,15]. Hence, it is essential to develop an alternative formulation of ibrutinib with improved oral bioavailability and higher efficacy.

During the last few years, nano-based drug carriers have been materialized as novel carriers due to their unique characteristics, such as the solubilisation effect, protection, modulation of drug release, and delivery of the drug at the specific target site, which resulted in enhanced antitumor activity of many difficult-to-formulate drugs while reducing the side effects [16]. A wide range of nanomaterials have been reported to enhance the drug delivery. It is essential to address the major challenges such as biocompatibility, permeation, drug loading, and toxicity during the development of nanocarriers. Due to these challenges, the focus on most effective nanocarriers such as liposomes has augmented significantly during few decades [17].

The nanoscale version of liposomes termed “nanoliposome” was recently explored with enhanced encapsulation efficiency and controlled delivery of bioactive molecules [18]. Because of the nanostructure, they markedly differ from conventional liposomes of the same composition in terms of physical, chemical, and biological properties [19]. It can be predicted that these nanocarriers can be effectively used for the delivery of bioactives because most of the biological processes occur at nanoscale. Nanoliposomes are composed of lipid and phospholipids and possess similar chemical, structural, and thermodynamic properties to that of conventional liposomes. Nanoliposomes are known to improve the technical attributes of final products and advance their biological functionalities [20,21]. The amphiphilic nature of nanoliposomes provide them with the ability to encapsulate both lipophilic and hydrophilic substances in their respective compartments [22]. These are promising carriers for the delivery of higher drug pay loads to the target site and prolong the circulation time in systemic circulation. The characteristic bilayer structure of these nanoliposomes is highly biocompatible, biodegradable, and most acceptable as a drug delivery vehicle. These nanocarriers can move through lipid bilayers of cancer cells with increased drug concentration at the site of action and eventually increase cytotoxicity [23,24]. Compared with other nano drug delivery systems, these nanoliposomes can be easily produced on a large scale using natural and inexpensive ingredients [25].

Despite the important benefits of nanoliposomes, further research is essential to address the destabilization and efficiency of liposomes. The components and formulation design are important factors to develop an efficient nanoliposome formulation with desired characteristics. The particle dimension of the liposomes is a prime character to consider, since a smaller particle size results in a greater surface area and consequently leads to better reactivity and controls drug release characteristics [26]. The entrapment efficiency of nanoliposomes is a crucial factor for promising applications due to the requisite to prepare a nanoformulation with an improved entrapment efficiency and minimal drug loss [27]. Finally, it is challenging to develop an economically viable method to prepare liposomes on a nanoscale with a high scalability and reproducibility [28].

The response surface approach encompasses a series of statistical and mathematical procedures for the building and exploitation of empirical models. By using the appropriate design and analysis of experiments, response surface methodology (RSM) relates a response parameter to the number of levels of input variables that influence it. RSM is a way of exploring the influence of operation factors on the response variables. RSM reduces the number of trails by using the multiple quadratic regression equation to fit the influencing factors and response variables. The optimum process parameters can be identified by analyzing the regression equation. The entire process could be conducted in three steps: design of experiments, assessment of the mathematical model, and prediction of the response value of the model [29]. Considering the above, RSM was used to obtain the optimum settings for the preparation of ibrutinib nanoliposomes.

The main aim of this work was to establish optimum conditions for the preparation of ibrutinib liposomes and to assess the stability of prepared nanoliposomes under different stress conditions.

## 2. Experimental

### 2.1. Materials

An ibrutinib sample was obtained from Honour lab Ltd., Hyderabad, India. L-α-phosphatidylcholine (from soybean), cholesterol, pepsin, and sterylamine were obtained from Sigma Aldrich (Bangalore, India). Chloroform and diethyl ether were bought from Innovative Chemical Interchange Pvt. Ltd., Hyderabad, India. Analytical grade chemicals and reagents were used throughout the study.

### 2.2. Methods

#### 2.2.1. Preparation of Ibrutinib Nanoliposomes

Ibrutinib nanoliposomes were prepared by using the reverse-phase evaporation method as reported elsewhere [30]. In the first step, ibrutinib, phosphatidylcholine, cholesterol, and sterylamine were dissolved in a mixture of solvents (diethyl ether and chloroform). In the second step, the organic solvents were removed by using a rotary evaporator under reduced pressure at 37 °C. Nitrogen gas was purged through the thin film for 10 min. The solvent residues were removed by keeping the sample in a vacuum oven at ambient temperature for 2 days. The resultant thin film was hydrated to form ibrutinib liposomes in 30 mL of phosphate buffer at 500 rpm using a mechanical stirrer for 30 min. The resulting dispersion was then subjected to ultrasonication at 180 watts and magnetic stirring to get homogenised nanoliposomes. This liposomal dispersion containing ibrutinib was stored in a freezer at 4 °C until further characterisation.

#### 2.2.2. HPLC Analysis

The quantitative estimation of ibrutinib was performed using the Agilent 1260 HPLC system (Agilent Technologies Inc., Santa Clara, CA, USA). A mixture of acetonitrile and water (30:70) was used as the mobile phase with flow rate 0f 0.6 mL/min. Ibrutinib was estimated at 258 nm using the Welch welchrom-C18 (250 × 4.6 mm, 5 μm) HPLC column at 25 °C. Empower-3 operating software was used for data recording and processing.

#### 2.2.3. Encapsulation Efficiency

Encapsulation efficiency of nanoliposomes was calculated by determining both bound and unbound drugs in the system. In the first step, the unbound drug was removed from the liposome by the dialysis method [31]. The amount of the drug that remained in the system after removal of the unbound drug was considered as the encapsulated drug. The ibrutinib formulation (5 mL ≈ 1 mg of drug) was placed into a dialysis bag (MWCO 12,000–14,000 da) and placed in 100 mL of pH 7.4 phosphate buffer. An amount of 1 M of sodium thiocyanate was added to the dialysis medium. The experiment was conducted at a temperature of 37 °C and a stirring speed of 100 rpm. A total of 2 mL of the sample was withdrawn from the dialysis media after 60 min to measure the amount of the unbound drug. The formulation that remained in the dialysis bag was dissolved in a mixture of water and organic solvent (methanol) to extract the bound drug from the formulation. The concentration of both the bound drug and the unbound drug were measured by the stability-indicating HPLC method. The percent encapsulation efficiency was estimated as per the Equation (1):

(1) $$Encapsulation\;efficiency=\frac{\left(Total\;amount\;of\;ibrutinib-Free\;ibrutinib\right)}{Total\;amount\;of\;ibrutinib}$$

#### 2.2.4. Determination of Particle Size and Zeta Potential

The particle size was determined by measuring the random change in intensity of light scattered from the nanoliposomal dispersion using the Malvern particle size analyser (Master sizer 2000). The samples were suitably diluted with Milli-Q water for every measurement. The measurements were made at a fixed angle of 90° for all samples. A diluted sample was used for the measurement of droplet size. The average droplet size and the polydispersity index were calculated using a cumulative analysis of the triplicate results. Zeta potential of the nanoliposomes was measured in an additional gold-plated electrode-containing U-shaped cell at a count rate of 250 particles/second at 25 °C. All the measurements were collected three times.

#### 2.2.5. Surface Morphology Observation by TEM

The JEOL JEM-2000FX transmission electron microscope was employed to check the surface morphology of ibrutinib nanoliposomes. One drop of aqueous solution of phosphotungstic acid was added to the diluted sample of the formulation for staining before observation under TEM.

#### 2.2.6. Design of Experiments

The formulation and process variables for the preparation of ibrutinib nanoliposomes were optimized by using RSM. Four variables, namely, the phosphatidylcholine-to-cholesterol ratio (A), conc. of ibrutinib (B), sonication time (C), and stirring time (D) were identified as main influencing factors based on the preliminary experiments conducted by varying a single factor at a time. A Box–Behnken design (BBD) with four factors at three levels was employed to optimize and evaluated the main, interactive, and quadratic effects of the influencing variables on the response parameters. The BBD is rotatable and requires at least three levels of each factor. The BBD is appropriate to build second-order polynomial models and to describe the quadratic response surfaces [32]. The range and level of each independent variable was identified based on the results of preliminary experiments (Table 1). As described by the BBD model, 29 experiments were randomly arranged by Design Expert^®^ software (V8.0.1, Stat-Ease Inc. Minnepolis). All the trial experiments were performed as per the design and the results are presented in Table 2.

#### 2.2.7. Data Analysis

The obtained results were subject to statistical analysis. The relationship between the variables can be described by using various models. Numerous statistical parameters such as the model *p*-value, the *p*-value of lack of fit, the regression coefficient (R^2^), the adjusted R^2^, and the coefficient of variation were considered to select a suitable best-fitting model. Usually, the model terms with *p*-values greater 0.005 can be considered as insignificant and can be eliminated from the model. Each response parameter can be evaluated by quadratic model using multiple regression analysis as shown in Equation (2):

(2) $$Y=A_{0}+A_{1}X_{1}+A_{2}X_{2}+A_{3}X_{3}+A_{4}X_{4}+A_{5}X_{1}^{2}+A_{6}X_{2}^{2}+A_{7}X_{3}^{2}+A_{8}X_{4}^{2}+A_{9}X_{1}X_{2}+A_{10}X_{1}X_{3}+A_{11}X_{1}X_{4}+A_{12}X_{2}X_{3}+A_{13}X_{2}X_{4}+A_{14}X_{3}X_{4}$$

where

Y—response parameter
A^0^—intercept
A^1^–A^14^—regression coefficients
X^1^, X^2^, X^3^ and X^4^—main influencing factors
X1×2—interactive effect
$X_{1}^{2},\;X_{2}^{2},\;X_{3}^{2}\;\&\;X_{4}^{2}$ —quadratic effect

The independent variables which do not contribute to the regression equation will be deleted one at a time by a backward elimination procedure. Three-dimensional response surface plots show the functional association between the selected response parameter and two independent variables. Perturbation and contour plots also can be used to visualize the influence of the independent variables on the response parameters [33,34].

#### 2.2.8. Optimization

The optimal points for the independent variables were attained using a numerical optimization technique by setting restrictions on the response parameters and influencing factors. The nanoformulation was prepared in triplicate under optimal conditions to verify the validity of the optimization technique.

#### 2.2.9. Stability of Ibrutinib Nanoliposomes

##### pH Stability

To assess the influence of pH on the stability of ibrutinib liposomes, 5 mL of the nanoformulation was added to phosphate buffer solutions of different pH values. The encapsulation efficiency of each sample was measured after 30 min [35]. The present release ratio of each sample was calculated using Formula (3).

(3) $$\%\;Release\;ratio=\left(1-\frac{\;EE_{t}}{EE_{s}}\right)\times 100\;\;\;\;\;\;\;\;\;\;\;\;\;\;\;\;$$

*EE_t_*—encapsulation efficiency of the test sample solution at a specific pH and
*EE_s_*—encapsulation efficiency of the standard sample at pH 7.

##### Thermal Stability

To assess the thermal stability, ibrutinib nanoliposomes samples were exposed to different temperature conditions (4, 25, 37, 60, and 80 °C) at a standard pH of 7 for 30 min. The percent encapsulation efficiency was calculated using Formula (1). The % release ratio of all the samples was calculated using Formula (4).

(4) $$\%\;Release\;ratio=\left(\frac{\;EE_{4}-EE_{T}}{EE_{s}}\right)\times 100\;\;\;\;\;\;\;\;\;\;\;$$

*EE_T_*—encapsulation efficiency of the test sample solution at a specific temperature and
*EE_s_*—encapsulation efficiency at the standard temperature (4 °C).

##### Effect of Ultrasound Time on Stability of Nanoliposomes

An 80-kHz cylindrical bath sonicator (MH, Mumbai, India) was used to generate the ultrasound. A 30 mL ibrutinib nanoliposome sample was subjected to ultrasonication in a bath sonicator, which is maintained at 4 °C. For every five-minute interval, 3 mL of sample was withdrawn, and the encapsulation efficiency was determined [35].

#### 2.2.10. Influence of Dissolution Medium on In Vitro Drug Dissolution

In vitro dissolution studies were conducted both in simulated gastric fluid and simulated intestinal fluid. A total of 5 mL of ibrutinib nanoformulation was placed in a one end sealed dialysis bag. An amount of 1 M of sodium salicylate was added to the dissolution medium so as to maintain the sink condition. At different time intervals (15, 30, 60, 120, 180, 240, 300, and 360 min), samples were withdrawn and replaced with same volume of fresh medium. The concentration of ibrutinib was estimated using the validated HPLC method. All the experiments were conducted in triplicate and the average results were reported.

#### 2.2.11. Fourier Transformed Infrared Spectroscopy

The FTIR spectra of plain ibrutinib, the physical mixture, and the nanoformulation were obtained by the KBr pellet method using an FTIR spectrophotometer (TENSOR 27, BRUKER Optics, Ettlingen, Germany) in the range of 400–4000 cm^−1^ at a resolution of 4 cm^−1^ with 99.999% nitrogen.

#### 2.2.12. Thermal Analysis

To confirm the possible interaction of ibrutinib with any of the excipients, differential scanning calorimetry studies (PerkinElmer DSC/7) were carried out for ibrutinib, the physical mixture, and the nanoformulation. The samples were subjected to gradual heating in the range of 30–400 °C under a nitrogen purge.

#### 2.2.13. X-ray Diffraction Study

XRD patterns of plain ibrutinib, the physical mixture, and the nanoformulation were recorded at a rate of 2°/min in the range of 10–60° on the Bruker D8 Advance X-ray diffractometer.

#### 2.2.14. Statistical Analysis

Design Expert^®^ software (Stat-Ease V8.0.1) was used to design and analyse the experimental results. A Box–Behnken design with 29 runs and 5 centre points were implemented to validate the polynomial equation by ANOVA. All the data obtained were expressed in terms of mean ± SD.

## 3. Results and Discussion

Several batches of ibrutinib nanoliposomes were prepared to understand the influence of the phosphatidylcholine-to-cholesterol ratio (A), conc. of ibrutinib (B), sonication time (C), and stirring time (D) on the drug encapsulation efficiency (Y_1_) and particle size (Y_2_). The reverse-phase evaporation method produced “inverted micelles”, which formed a gel-like viscous structure when the organic solvents were removed. BBD was used to optimize the process and formulation variables so as to obtain ibrutinib nanoliposomes with an enhanced encapsulation efficiency and a reduced particle size. The selected model was found to be significant with respect to the encapsulation efficiency and particle size as designated by the corresponding ‘*p*’ values (*p* < 0.05).

### 3.1. RSM Optimization

#### 3.1.1. Statistical Treatment

Sequences of twenty-nine trials were executed as per a four-factor, three-level BBD. The results obtained from the randomized experiments are presented in Table 2. Response surface methodology was employed to assess the affiliation among the independent variables and to predict the optimal conditions essential to yield an exact anticipated response. Typically, RSM methodologies reduce the number of experimental runs and appraise the interaction effects between selected variables. Resultant data was analysed to find ANOVA values, regression coefficients, and the regression equation. All the results were fitted into a second-order quadratic model and the appropriateness of the model was confirmed by analysis of variance, lack of fit, and the regression coefficient (R^2^) values. The highest F-value for both the quadratic models indicated the best fitting models as presented in Table 3.

Mathematical equations for both the response variables were generated by means of multiple linear regression analysis and are presented in Table 4. The relative magnitudes of coefficients of individual independent variables are associated to the individual effect on the response variables. The interactive and quadratic effects of the independent variables were evident from the coefficients with more than one variable term and higher order terms, respectively. Coefficients with a positive sign indicate synergistic effects and those with a negative sign indicate antagonistic effects. Significant lack of fit indicates inefficiency of the prediction model, hence a model with significant lack of fit is not recommended. Both the quadratic models displayed a non-significant lack of fit, demonstrating the fitness of the model.

Multiple regression analysis for both the models is shown in terms of the R^2^ values, the adjusted R^2^ value, and the coefficient of variation. An R^2^ value is a measure of variation around the mean. The R^2^ values for both the response parameters were greater than 0.98, demonstrating the appropriateness of the model. The adjusted R^2^ value is another essential parameter to indicate the adequacy of the model. Higher values of R^2^ do not always indicate the adequacy of the model because the other variable terms also will contribute to the higher R^2^ values. Hence, it is important to consider the adjusted R^2^ value also to appraise model adequacy. The regression coefficient (R^2^) values for both Y_1_ and Y_2_ are 0.9877 and 0.9970, respectively. Both the R^2^ and the adjusted R^2^ values for both the models did not vary significantly, indicating that the non-significant terms in the model have been eliminated. The coefficient of variation values for both the responses were found to be 1.45 and 1.22, which indicated the reproducibility and reliability of the obtained results.

**Figure 1:**
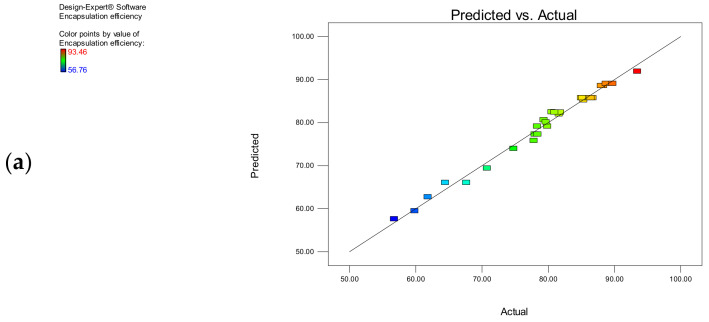

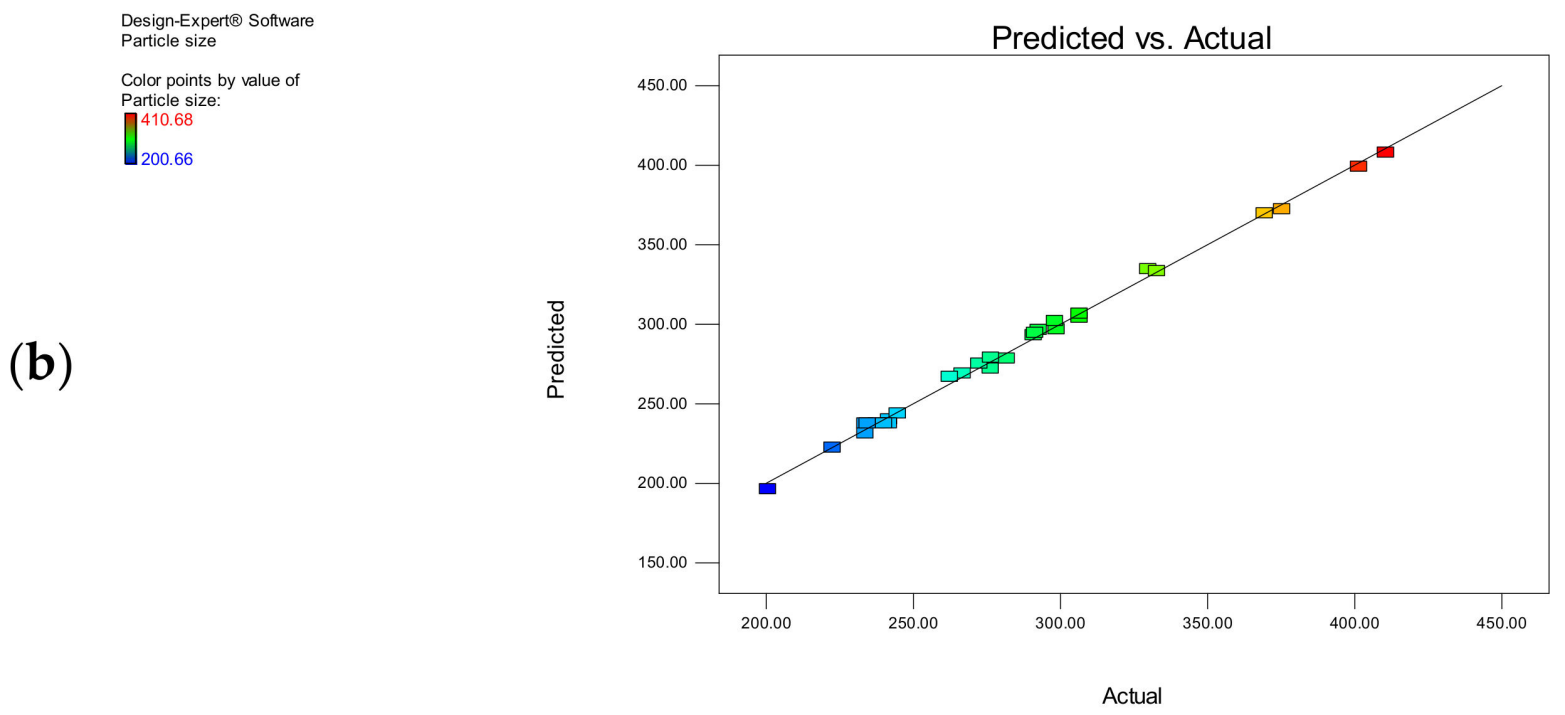
Comparison between predicted and actual values for (**a**) encapsulation efficiency and (**b**) particle size.

Figure 1a,b display the reasonably good correlation between predicted and actual results. The results indicated that both the models were able to recognize the process and formulation variables for the preparation of ibrutinib nanoliposomes.

#### 3.1.2. Encapsulation Efficiency

Encapsulation of the drug in the nanoliposomes can be important for enhancing the oral bioavailability of the drug. Hence, the first part of the study was aimed to investigate the factors affecting drug encapsulation in the nanoliposomal formulation. The encapsulation efficiency of nanoliposomes was found to be in the range of 56.76% to 93.46% (Table 2). The polynomial model has shown that the factors A, B, and C have a significant effect on encapsulation efficiency.

**Figure 2:**
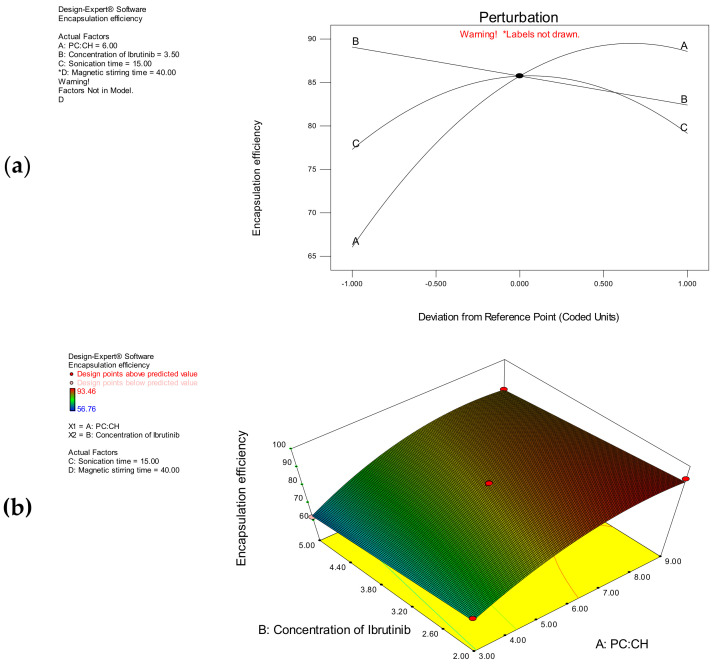
(**a**) Two – dimensional perturbation plot showing the effect of A, B, and C on the encapsulation efficiency; (**b**) 3D response surface plot showing the interactive effect of A and B on the encapsulation efficiency at constant level of C and D. (* – indicates the Factor D have not shown any significant influence on encapsulation efficiency).

The mathematical model of the encapsulation efficiency was found to be significant with a model F-value of 370.06. The model terms A, B, C, A^2^, and C^2^ were found to be significant with *p*-values less than 0.0500. The “lack of fit F-value” (2.52) suggests that it is not significant. There is a 19.18% chance that this large value could occur due to noise. The non-significant lack of fit is good to describe the model fitness. It is evident from the factorial equation that the variable A has a more significant influence on the encapsulation efficiency than B and C, whereas the variable D has been eliminated from the model as it has no significant effect. The regression coefficient (R^2^) and the adjusted R^2^ values for the model are 0.9877 and 0.9853, respectively. The effect of individual variables on the encapsulation efficiency was described by using a perturbation plot (Figure 2a). The variable A has the main effect on the encapsulation efficiency followed by B and C, which have a moderate and a little effect, respectively. A three-dimensional response surface plot was used to explain the interactive effect of A and B (AB) at constant levels of C and D (Figure 2b).

As the phosphatidylcholine-to-cholesterol ratio (A) increases, the encapsulation efficiency increased from 56.76 to 93.46%. The increased proportion of cholesterol might have changed the order of mobility of ibrutinib in the lipid bilayer. The encapsulation efficiency was increased from 79.65 to 93.46 at lower values of A. At higher values of A, an antagonistic quadratic effect was observed. A higher encapsulation efficiency was observed at lower concentrations of ibrutinib. The encapsulation efficiency was increased from 70.77 to 93.46% at lower concentrations of ibrutinib. In contrary to this, the encapsulation efficiency decreased from 85.34 to 61.84% at higher concentrations of ibrutinib. The sonication time enhanced the encapsulation efficiency at low values, and higher levels decreased the encapsulation efficiency. At low values of C, the encapsulation efficiency increased from 56.76 to 79.65%. At high levels of C, the encapsulation efficiency decreased from 81.62 to 59.84%. The stirring time has not influenced the encapsulation efficiency significantly.

#### 3.1.3. Particle Size

Particle size determination is an important quality control measure to measure the ability of any nanoformulation. Size distribution is significant in terms of stability, solubility, dissolution, and permeation through various tumor tissues and organs [36]. The particle size of the nanoliposomes was found to be in the range of 200.56 to 410.68 nm as presents in Table 2. The polynomial model shows that all the variables (A, B, C, and D) have a significant effect on the particle size of nanoliposomes.

**Figure 3:**
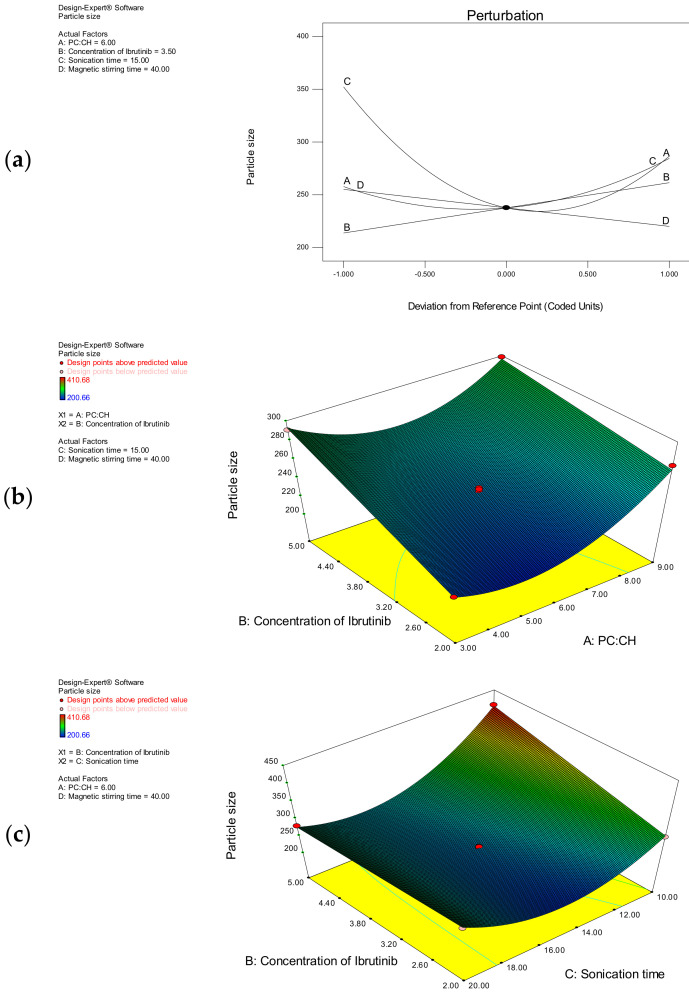
(**a**) Two – dimensional perturbation plot showing the effects of A, B, C, and D on the particle size; (**b**) 3D response surface plot showing the interactive effect of A and B on the particle size at constant levels of C and D; (**c**) 3D response surface plot showing the interactive effect of B and C on the particle size at constant levels of A and D.

The mathematical model of the particle size was found to be significant with a model F-value of 817.93. The model terms A, B, C, D, AB, BC, A^2^, and C^2^ were found to be significant with *p*-values less than 0.0500. The “lack of fit F-value” (1.04) suggests the non-significant lack of fit. There is a 54.38% chance that this large value could occur due to noise. The non-significant lack of fit is good to describe the model fitness. It is evident from the equation that the variable C has more a significant influence on the particle size than other variables. The regression coefficient (R^2^) and the adjusted R^2^ values for the model were 0.9970 and 0.9857, respectively. The effect of individual variables on the particle size was described by using a perturbation plot (Figure 3a). The variable C has the main and major effect followed by B, D, and A, which have moderate effects on the particle size. Three-dimensional response surface plots were used to describe the interactive effect of independent variables. The interactive effect of AB on the particle size at constant levels of C and D is as shown in Figure 3b. Similarly, the interactive effect of BC on the particle size at constant levels of A and D is as shown in Figure 3c.

As the concentration of ibrutinib increased, the particle size was increased. Likewise, as the concentration of cholesterol increased, the particle size was increased. The particle size was reduced at lower values of C, and higher values of C had an antagonist effect. The particle size was reduced as the stirring time was increased. As the phosphatidylcholine-to-cholesterol ratio (A) increased, the particle size increased from 222.56 to 401.52 nm. The increased proportion of cholesterol might have resulted in the increased particle size. At low levels of A, the particle size increased from 222.56 to 375.38 nm. Similarly, at high levels of A, the particle size increased from 262.46 to 401.52 nm. At higher levels of A, a synergistic quadratic effect was observed. Ibrutinib concentration has a positive effect on the particle size. At low levels of B, the particle size increased from 200.66 to 292.63 nm. Similarly, at high levels of B, the particle size increased from 244.78 to 410.68 nm. The sonication time and stirring time have antagonistic effects on the particle size. As the sonication time increases, a gradual decrease in particle size was observed. At the same time, when the preparation was exposed to sonication for longer periods, nanoparticles with increased dimensions were observed. This can be attributed to the rupture of the liposomal structure at higher sonication times. At low levels of C, the particle size decreased from 410.68 to 292.63 nm. In the same way, at high levels of C, the particle size decreased from 332.88 to 266.82 nm. At low levels of D, the particle size decreased from 369.48 to 233.76 nm. Correspondingly, at high levels of D, the particle size decreased from 329.92 to 200.66 nm.

### 3.2. Optimization

Derringer’s desirability function (D) was used to optimize the selected variables which influence the response parameters. Both the responses (encapsulation efficiency and particle size) were transformed into a desirability scale. Y_max_ and Y_min_ were considered as the objective function (D) for each response parameter. At last, each individual desirability function was merged as a function of geometric mean by an extensive grid and feasibility search over the domain to obtain the global desirability value using the Design Expert^®^ software. The extreme desirability function value was obtained at A: 6.76, B: 2.00, C: 15.13, and D: 45 min, with the confirming D value of 0.962. To confirm the appropriateness of the model, three executive batches of nanoliposomes were prepared under optimal conditions. The response parameters for the prepared batches are as shown in Table 5. A close agreement between the predicted and the experimental values demonstrates the validity of the experimental design (BBD) combined with Derringer’s desirability function for the optimization of ibrutinib nanoliposomes.

**Figure 4:**
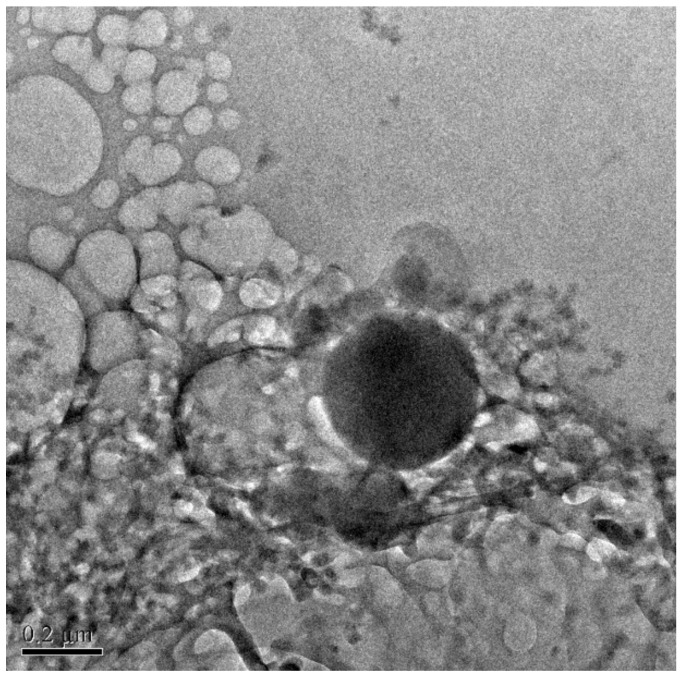
TEM image of ibrutinib nanoliposomes.

The mean particle size, polydispersity index, and zeta potential values of the prepared batches were as shown in Table 6. TEM images revealed well-formed, spherical, and unilamellar vesicles in the size range of 200–250 nm (Figure 4). Moreover, the particle size determined by the light scattering method is in concurrence with the TEM measurement.

### 3.3. Stability of Ibrutinib Nanoliposomes

#### 3.3.1. pH Stability

**Figure 5:**
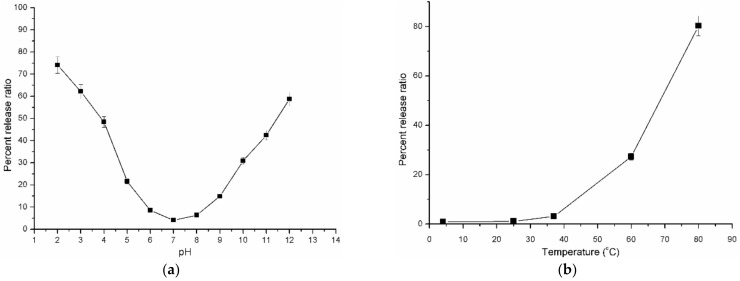
(**a**) Influence of pH on the percent release ratio of nanoliposomes; (**b**) effect of temperature on the drug release ratio of nanoliposomes.

Different buffer solutions with a range of pH values (2 to 12) were used to assess the stability of the prepared nanoliposomes. The percent release ratio of the drug from nanoliposomes dissolved in phosphate buffers of different pHs is as displayed in Figure 5a. The results specified that the pH of buffer solution had a great influence on the percent release of the drug from the nanoformulation. The percent release ratio of ibrutinib at a pH of 2 and 12 was 74% and 59%, respectively. A significant decrease in the release ratio was observed with a drop in pH values until a pH of 7 from both the extremes. The percent release of ibrutinib is around 4% at a pH of 7, indicating that the nanoliposomes will be stable under a neutral environment.

#### 3.3.2. Thermal Stability

Five samples of ibrutinib nanoliposomes were exposed to altered temperatures (4, 25, 37, 60, and 80 °C) for 30 min. The effect of temperature on the percent release ratio of all the samples is as revealed in Figure 5b. The results evidently showed that ibrutinib nanoliposomes are comparatively stable at 4–40 °C. The highest release of the drug (~80%) at an elevated temperature (80 °C) was observed due to the rupture of the liposomal formulation. As the temperature increases, the release rate was increased. Hence, it is essential to maintain the preparation at lower temperatures.

#### 3.3.3. Effect of Ultrasound Time on the Stability of Nanoliposomes

**Figure 6:**
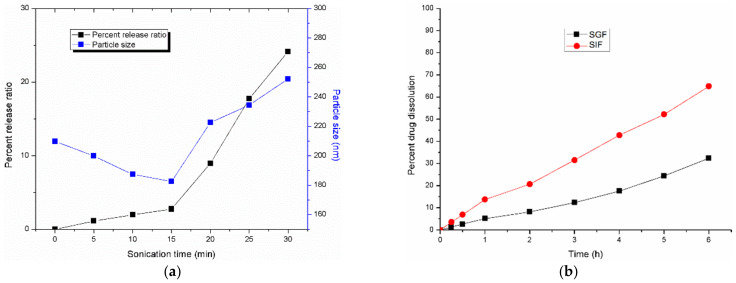
(**a**) Effect of ultrasound on the stability of ibrutinib nanoliposomes; (**b**) effect of dissolution medium on the in vitro release of ibrutinib nanoliposomes.

Ultrasonication is an effective method to obtain the nanoformulation with a homogeneous particle size distribution. In this study, the influence of ultrasound on the drug release ratio was evaluated. The results indicated that the ultrasound accelerated the release of the drug from the nanoformulation after 15 min. As the exposure time increased, the drug release ratio was increased as shown in Figure 6a. Initial exposure for the first fifteen minutes resulted in a decrease in particle size, and the release ratio of the drug was found to be minimal. The release ratio was found to be around ~24% after ultrasound exposure for 30 min, which indicated the outflow of the drug from the nanoliposomes. From the results, it can be suggested that the ultrasound-assisted drug release can be promising for further research. From the results, it is understood that ultrasound treatment for 15 min may be suitable to reduce the particle size.

### 3.4. In Vitro Dissolution Study

The dissolution pattern of ibrutinib from nanoliposomes is conducted both in simulated intestinal fluid (SIF) and simulated gastric fluid (SGF) to assess the stability of the drug in both media. It is important to assess the shielding effect of the lipid layer on the core materials due to the usual degradation of the drug by the higher concentrations of acids and enzymes present in SGF. Figure 6b indicated that about 32% of ibrutinib was released from the nanoformulation at 6 h in SGF. The lipid layer is capable of protecting the drug in SGF, whereas in SIF, around 65% of the drug was released at 6 h. In both the media, a slow and sustained release pattern of the drug was witnessed. The results collectively indicated the stability of the preparation in the stomach and the controlled release in the intestine.

### 3.5. FTIR Spectra

**Figure 7:**
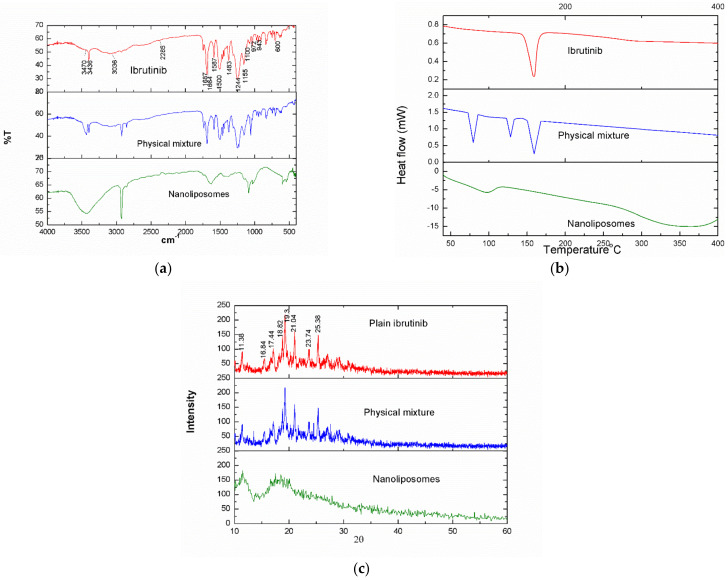
(**a**) FTIR spectra of the plain ibrutinib, the physical mixture, and the nanoformulation; (**b**) DSC thermogram of the plain ibrutinib, the physical mixture, and the nanoformulation; (**c**) XRPD pattern of the plain ibrutinib, the physical mixture, and the nanoformulation.

Figure 7a shows the FTIR spectra of ibrutinib, the physical mixture, and the nanoliposomes. FTIR spectra of the plain drug shows several characteristic peaks: 3470 cm^−1^ and 3436 cm^−1^ (N-H stretching vibrations), 3036 cm^−1^ (aromatic C-H stretching vibration), 1664 cm^−1^ to 1613 cm^−1^ (C=O stretching), 1587 cm^−1^ and 1500 cm^−1^ (aromatic C=C and C=N vibrations), and 1483 to 600 cm^−1^ (bending frequencies of HCN and HCH in addition to peaks of υCH, υCC, υOC, and υNC). The characteristic peaks of the drug are in concurrence with the reported values [37,38,39]. All the major characteristic peaks of the drug were observed with the FTIR spectra of the physical mixture along with the additional peaks of excipients. In the FTIR spectra of the nanoformulation, all the major characteristic peaks of ibrutinib were observed with broadening. This might be attributed to the possible interaction of ibrutinib with the other components of the formulation.

### 3.6. DSC Thermograms

Differential scanning calorimetry curves of the plain ibrutinib, the physical mixture, and the ibrutinib nanoformulation are displayed in Figure 7b. The DSC thermogram of ibrutinib shows a sharp endothermic peak corresponding to its melting point at 159 °C. The DSC curve of the physical mixture shows different endothermic peaks corresponding to each individual component. The characteristic peak of the drug was not altered in the DSC curve of the physical mixture, indicating the absence of interactions with the excipients, whereas the characteristic peak of the drug was not observed in the nanoformulation. This can be attributed to the interaction between the components of the formulation and the homogenous dispersion of the drug in the lipid.

### 3.7. X-ray Diffraction Pattern

The specific intense characteristic peaks displayed at the 2θ values of 10.6, 11.38, 16.84, 17.44, 18.82, 19.3, 21.04, 23.74, and 25.38° indicate the crystalline nature of the plain ibrutinib as shown in Figure 7c. The observed peaks are similar to the reported peaks [37]. The same characteristic crystalline peaks of the drug were observed in the physical mixture, indicating the compatibility of drug with excipients whereas, the characteristic crystalline peaks of the drug disappeared in the spectra of the nanoformulation, indicating the amorphization of the drug in the lipid structure.

## 4. Conclusions

The effects of the selected four variables for the preparation of ibrutinib nanoliposomes were investigated using response surface methodology. The finest parameters were obtained using a numerical optimization technique with a desirability function of 0.962. Statistical analysis of the data revealed the main, interactive, and quadratic effects of the independent variables on the response parameters. Confirmation experiments performed at the optimum conditions revealed the reliability of the optimization technique for the preparation of nanoliposomes. Stability studies revealed the suitable conditions for keeping the nanoliposomes. The ultrasound-assisted release pattern of the drug can be promising for further studies.

## Figures and Tables

**Table 1:** BBD with list of dependent and independent variables.

Independent Variables			Levels		
Variable		Units	Low	Intermediate	High
A	PC: CH	*w*/*w*	3	6	9
B	Conc. Ibrutinib	*w*/*v*	2	3.5	5
C	Sonication time	min	10	15	20
D	Stirring time	Min	35	40	45
**Dependent variables**			**Goal**		
Y_1_	Encapsulation efficiecny	%	Increase		
Y_2_	Particle size	nm	Decrease		

**Table 2:** Observed responses of trial experiments as per BBD.

Exp.	A	B	C	D	Y_1_	Y_2_
1	6	3.5	20	35	78.34 ± 2.78	306.56 ± 5.12
2	3	3.5	10	40	56.76 ± 0.86	375.38 ± 6.34
3	9	2	15	40	93.46 ± 0.93	276.34 ± 5.51
4	6	3.5	15	40	86.42 ± 4.08	234.56 ± 6.18
5	3	5	15	40	61.84 ± 2.87	290.92 ± 5.42
6	6	3.5	15	40	85.82 ± 2.54	241.72 ± 4.86
7	6	3.5	15	40	86.72 ± 3.32	237.64 ± 4.63
8	6	5	20	40	77.83 ± 4.08	281.78 ± 5.17
9	6	2	15	45	89.73 ± 0.78	200.66 ± 4.78
10	6	3.5	15	40	84.98 ± 2.42	233.78 ± 3.92
11	6	2	20	40	80.54± 1.28	291.42 ± 6.13
12	3	2	15	40	70.77 ± 1.44	222.56 ± 2.97
13	6	5	15	35	81.82 ± 1.56	276.42 ± 3.93
14	6	2	15	35	88.72 ± 4.18	233.76 ± 4.12
15	9	3.5	20	40	81.62 ± 3.18	332.88 ± 2.77
16	9	3.5	15	35	88.34 ± 3.76	298.18 ± 4.15
17	9	3.5	15	45	87.98 ± 4.12	262.46 ± 4.75
18	6	3.5	10	35	77.98 ± 2.12	369.48 ± 5.15
19	6	3.5	20	45	79.88 ± 3.38	266.82 ± 6.19
20	9	5	15	40	85.34 ± 1.98	298.72 ± 4.36
21	3	3.5	15	35	64.46 ± 2.24	272.44 ± 5.27
22	6	3.5	10	45	78.42 ± 1.32	329.92 ± 6.22
23	6	5	10	40	74.78 ± 1.76	410.68 ± 3.18
24	9	3.5	10	40	79.65 ± 3.42	401.52 ± 4.27
25	3	3.5	20	40	59.84 ± 2.56	306.56 ± 5.16
26	6	3.5	15	40	85.14 ± 2.26	240.12 ± 3.38
27	3	3.5	15	45	67.66 ± 1.98	241.82 ± 3.92
28	6	5	15	45	80.96 ± 3.12	244.78 ± 2.96
29	6	2	10	40	79.34 ± 2.67	292.63 ± 3.18

**Table 3:** Analysis of variance of both the quadratic models.

Source of Variation	Sum of Squares	Degrees of Freedom	Mean Square Value	F-Value	*p*-Value Prob > F
***Y*_1_ *—Encapsulation efficiency***					
Model	2437.769	5	487.5538	370.0586	<0.0001
A—PC:CH	1520.1	1	1520.1	1153.773	<0.0001
B—Concentration of ibrutinib	133.2667	1	133.2667	101.1509	<0.0001
C—Sonication time	10.30453	1	10.30453	7.821253	0.0102
A^2^	489.6346	1	489.6346	371.638	<0.0001
C^2^	389.8075	1	389.8075	295.8681	<0.0001
Residual	30.3026	23	1.317504		
Lack of fit	27.96468	19	1.471825	2.518179	0.1918
Pure error	2.33792	4	0.58448		
Total	2468.07	28			
Observed R^2^	0.9877				
Adjusted R^2^	0.9851				
CV	1.45				
***Y*** **_2_** **—** ***Particle size***					
Model	79,741.52	8	9967.69	817.9295	<0.0001
A—PC:CH	2144.548	1	2144.548	175.9775	<0.0001
B—Concentration of ibrutinib	6812.997	1	6812.997	559.0614	<0.0001
C—Sonication time	12,909.42	1	12,909.42	1059.323	<0.0001
D—Stirring time	3688.312	1	3688.312	302.6558	<0.0001
AB	528.5401	1	528.5401	43.37098	<0.0001
AC	4076.184	1	4076.184	334.4838	<0.0001
A^2^	7740.406	1	7740.406	635.1628	<0.0001
C^2^	46,092.1	1	46,092.1	3782.229	<0.0001
Residual	243.7298	20	12.18649		
Lack of fit	196.5759	16	12.28599	1.042203	0.5438
Pure error	47.15392	4	11.78848		
Total	79,985.25	28			
Observed R^2^	0.9970				
Adjusted R^2^	0.9857				
CV	1.22				

**Table 4:** Polynomial equation for the responses—encapsulation efficiency and particle size.

Dependent Variable	Regression Equation
Encapsulation efficiency (Y_1_)	85.74 + 11.25 A − 3.33B + 0.93 C − 8.42 A^2^ − 7.52 C^2^
Particle size (Y_2_)	237.59 + 13.37 A + 23.83 B − 32.80 C − 17.53 D − 11.49 AB − 31.92 BC + 33.49 A^2^ + 81.72 C^2^

**Table 5:** Optimum conditions attained by applying restrictions on the response parameters.

Independent Variables	Optimum Values	Predicted Values		Actual Values		
		Y_1_	Y_2_	Batch	Y_1_	Y_2_
A	6.76 (*w*/*w*)	91.39	204.67	1	89.94 ± 1.76	208.34 ± 2.42
B	2% *w*/*v*			2	91.22 ± 2.12	211.76 ± 1.32
C	15.13 min			3	90.86 ± 3.27	205.42 ± 3.14
D	45 min					

**Table 6:** Particle size, polydispersity index, and zeta potential values.

Batch	Particle Size (nm)	Polydispersity Index	Zeta Potential (mV)
1	208.34 ± 2.42	0.212 ± 0.005	18.72 ± 3.18
2	211.76 ± 1.32	0.192 ± 0.005	20.43 ± 1.14
3	205.42 ± 3.14	0.234 ± 0.005	21.12 ± 1.18

## Data Availability

Not applicable.
